# Food Captures Attention, but Not the Eyes: An Eye-Tracking Study on Mindset and BMI’s Impact on Attentional Capture by High-Caloric Visual Food Stimuli

**DOI:** 10.5334/joc.210

**Published:** 2022-02-21

**Authors:** Leonardo Pimpini, Sarah Kochs, Wieske van Zoest, Anita Jansen, Anne Roefs

**Affiliations:** 1 Clinical Psychological Science, Maastricht University, Maastricht, NL; 2 School of Psychology, University of Birmingham, Birmingham, GB

**Keywords:** Attentional bias, Food intake, Mindset, Obesity, Eye-tracking, Bogus taste test

## Abstract

Obesity is a worldwide pandemic and theories propose that attentional bias (AB) for food triggers craving and overeating, especially for people with obesity. However, empirical evidence is inconsistent, which may be due to methodological diversity and the double-sided nature of high-caloric palatable foods. That is, these foods simultaneously have a high hedonic and a low health value. So, depending on context and/or emotional state, people’s *mindset* while viewing foods may alternate between hedonic (taste) and health (calories) values, possibly affecting AB for food in opposite directions. This study tests how mindset and BMI (Body Mass Index) influences AB and food intake. We expect greater AB for food and more food intake in the hedonic compared to the health mindset, especially for people with obesity. Mindsets were induced using short video-clips in two sessions in counterbalanced order. Participants (35 with a healthy-weight-category BMI, 31 with obesity) performed a modified *Additional Singleton* paradigm where they searched for a neutral target among neutral fillers. On 90% of the trials, either a food or a neutral distractor appeared. Response latencies to the target and eye-movements to the distractor were recorded. Dependent variables included: response latencies, and eye-movement variables on the distractor: fixations (%), 1st fixation duration, dwell-time. Food intake was assessed in a bogus taste test. No significant effects emerged from the eye-movements analysis, whereas the analysis of response latencies showed an AB for food, not significantly moderated by BMI or mindset. Food intake was affected by mindset partly as expected, as participants ate more in the hedonic than in the health mindset when the hedonic mindset was induced in the second session. One AB measure (fixations) correlated positively with food intake. Finally, food captured attention – but not the eyes – and mindset affects food intake partly as expected.

## Introduction

In today’s *obesogenic* environment, it is a challenge to maintain a healthy body weight, as we are constantly surrounded by high-caloric palatable foods, especially in the western world (Chaput et al., 2011; Townshend & Lake, 2017). However, not everyone is equally affected by the obesogenic environment. One factor that may explain this differential susceptibility to this environment is thought to be attentional bias (AB) for high-caloric foods (Hendrikse et al., 2015; Hetherington & Cecil, 2010; Llewellyn & Wardle, 2015; Werthmann et al., 2015; Yokum et al., 2011). AB for food is defined as increased attention for food stimuli relative to other stimuli. It is theorized that an AB for food elicits and maintains craving and can thereby lead to overeating and eventually weight gain (Field et al., 2016a; Hendrikse et al., 2015; Nijs, Muris, et al., 2010; Nijs & Franken, 2012a). Based on this theory, the hypothesis has been put forward that people with overweight and obesity show a stronger AB for food as compared to people with a healthy-weight-category BMI (Doolan et al., 2014; Robinson & Berridge, 1993). In the present study we build on this research, and we test how BMI, in interaction with participants’ current mindset (hedonic vs. health), influences AB for high-caloric foods. We expect a stronger AB for food in the hedonic as compared to the health mindset, and we expect this effect to be most pronounced in people with obesity.

Over the last 15 years approximately, many studies have tested the hypothesis that people with overweight and obesity have a stronger AB for high-caloric foods as compared to people with a healthy-weight-category BMI (e.g., Castellanos et al., 2009; Hendrikse et al., 2015; Volkow et al., 2011; Yokum et al., 2011). Particularly it has been stated that, to some extent, drugs and food activate common reward circuitry in the brain, therefore, drug-studies represents an insightful bridge to better understand food-related behaviours (e.g., Davis & Carter, 2009; Pelchat et al., 2004; N. D. Volkow et al., 2011; Nora D. Volkow et al., 2008; Nora D. Volkow & Wise, 2005; G. J. Wang et al., 2004, 2009). As a consequence, the so-called the ‘addiction model of obesity’ has gained much popularity (Nijs et al., 2010; Nijs & Franken, 2012; Volkow et al., 2011; Volkow & Wise, 2005; G. J. Wang et al., 2002; 2009). That is, similar to drug-related addiction, AB for high-caloric palatable foods might play an important role in the development and maintenance of (over)eating behaviours and weight gain (Volkow & Wise, 2005). Similarly, a recent ‘temptation management’ model of obesity treatment proposed that high caloric palatable foods may act as ‘motivational magnets’ monopolizing attention and provoking dietary lapses and weight regain (Appelhans et al., 2016; Field et al., 2016).

Results of some of these studies are in line with this hypothesis, and found evidence for AB for high-caloric food specifically in people with overweight (Batterink et al., 2010; Castellanos et al., 2009; Hendrikse et al., 2015; Yokum et al., 2011). However, results from other studies suggest that attentional avoidance of high-caloric foods is associated with increased BMI (Favieri et al., 2020; Nummenmaa et al., 2011; Veenstra et al., 2010; Veenstra & de Jong, 2012), and still other studies observed an approach-avoidance pattern of AB for high-caloric food (Deluchi et al., 2017; Kemps et al., 2013; Werthmann et al., 2011). Finally, a number of studies reported no significant differences in AB for high-caloric food between people with overweight/obesity and people with healthy-weight-category BMI (Doolan et al., 2014; Loeber et al., 2012; Nijs, Muris, et al., 2010; Nijs & Franken, 2012b). Taken together, the pattern of results from studies investigating AB for high-caloric food is highly variable and inconsistent (Doolan et al., 2014; Field et al., 2016; Roefs et al., 2015; Werthmann et al., 2015). These conflicting findings may be due to factors such as the use of different methodological approaches to measure AB for food (e.g., Stroop-task and the visual dot-probe task), hunger level, caloric content of food stimuli (Cunningham & Egeth, 2018), and individual eating style traits (Doolan et al., 2014).

To date, most studies have used either the Stroop paradigm (e.g., Dobson & Dozois, 2004; Hollitt et al., 2010; Pothos et al., 2009; Wilson & Wallis, 2013) or the visual dot-probe paradigm (e.g., Kemps et al., 2014; Nijs & Franken, 2012; Werthmann et al., 2015) to investigate AB for food. In the modified Stroop task, words are presented one at a time in different colors, and participants are required to identify the color of the words while ignoring their meaning. If participants are slower to identify the color of one category of words (e.g., food-related words) than another (e.g., office related words), it is concluded that attention is biased towards the former category of words. In the visual probe task, two pictures are simultaneously presented in the centre of the screen: one slightly to the left, the other slightly to the right. After the presentation of the stimuli, on average 2000 ms, a visual ‘probe’ (e.g., a dot) appears in the position of one of the two pictures (i.e., left or right). Participants are instructed to respond to the probe as rapidly and accurately as possible by pressing a button (left or right). If the average response latency is faster to probes that replace one type of stimulus (e.g., food) compared to another (e.g., office supplier), it is concluded that attention is biased towards the former type of stimulus (e.g., Mogg et al., 1998; Yiend, 2010).

Importantly, these two paradigms may lack ecological validity, may not really reflect food-related situations in daily life, in which often many stimuli are present at once. For instance, while reading the digital version of one’s favourite magazine, people can easily get distracted by a pop-up advertisement of a high-caloric palatable snack. The snack food is completely irrelevant for the task at hand, that is, reading the magazine. Therefore, we need a paradigm that involves visual competition (multiple stimuli appearing at once, instead of one or two at a time) and tests the attention-grabbing power of food while people perform a neutral task, such as reading a magazine.

In an effort to increase ecological validity, a modified ‘Additional Singleton’ paradigm (ASP) is adopted in the present study (Theeuwes, 1992; Theeuwes et al., 1998). To our knowledge, the present study is one of the very few studies (Cunningham & Egeth, 2018; Nummenmaa et al., 2011; Schmidt et al., 2016) investigating the power of food to capture attention and eye-movements while participants perform a neutral visual search task. Furthermore, the distractor stimuli (food vs. neutral) are entirely task-irrelevant and are displayed outside central vision, whereas in the often-used dot-probe task, food stimuli are presented in central vision. Thus, in this setting, for the distractors to be identified and processed, explicit eye-movements are required.

More specifically, in this modified version of the ASP participants are asked to search for a neutral target (the only grey circle) surrounded by neutral fillers (red circles). On 90% of the trials, a salient distractor (either a high-caloric food or a musical instrument) abruptly appears on screen. As soon as the target is selected with their eyes, participants are asked to press a button according to the letter (‘C’ or ‘reversed C’) inside the target. Previous work (Theeuwes et al., 1998; Theeuwes & Godijn, 2002) demonstrated that an abrupt onset of a salient task-irrelevant distractor automatically and involuntarily interferes with goal-directed search behavior. Therefore, we propose that this paradigm is able to better mirror daily life situations, comparable to the situation where you are driving on the highway and the golden arches of a famous fast-food chain automatically capture your attention.

Moreover, we do not simply look at foods in isolation, instead, we often get distracted by (high-caloric) foods while performing another task. Just like when, in the supermarket, the bright packaging of our favorite snack enters our visual field while searching for a piece of fruit or vegetables.

In addition to the diversity in methodology to assess AB for food, an often-overlooked explanation for the variable pattern of results is the double-sided nature of high-caloric foods. That is, on the one hand these foods have a high hedonic value, whereas, on the other hand, they have a low health value as overconsumption of these may lead to weight gain. So, the way people perceive (high-caloric) foods may depend on their current mindset. That is, people might be in a hedonic or health mindset depending on, for example, mood, situation, and/or context (Roefs et al., 2015, 2018; Werthmann et al., 2015). To introduce the idea of mindset, just imagine how you would ‘look’ at your favourite slice of chocolate cake at your friend’s birthday dinner, at the end of a tiring week of work. Now, instead, try to imagine the same slice of chocolate cake while walking through the hall of a fitness gym, or leafing through the pages of a women’s magazine.

A number of studies have provided evidence for the idea that mindset (either hedonic or health) affects AB for food as well as craving and intake (for a review, Roefs et al., 2018). In this recent review, it is suggested that AB for food should not be considered as a static BMI-related trait, but rather as a dynamic state, strongly dependent on people current mindset. In line with this idea, Werthmann et al. (2016), using the dot-probe task, found that AB for high-caloric food was attenuated in a health as compared to a hedonic mindset, specifically in people scoring high on dietary restraint. Similarly, two recent studies (Liu, Nederkoorn, et al., 2019a; Liu, Roefs, et al., 2019b) investigated AB for food using the same dot-probe paradigm, but a novel computing method to analyse the data: the Trial Level Bias Score (TL-BS) (Zvielli et al., 2015, 2016). The idea behind this method is to study AB over time, focusing on AB towards and away from food stimuli over the course of the experimental task. Results from the two studies (Liu et al., 2019a, 2019b) showed a significant positive correlation between BMI and food-related TL-BS, which reflects the degree of variability in AB for food. So, a higher BMI was associated with stronger variability in AB for food, more fluctuation in AB towards and away from food.

Similarly, the effect of mindset also extends to brain activity in response to visual food stimuli (Franssen et al., 2020). Results showed that brain activity was larger – including in several regions of the mesocorticolimbic system – with a hedonic attentional focus as compared to a neutral attentional focus, independently of the palatability of the visually presented food stimuli. These findings suggest that the level of brain activity reflects motivational saliency, rather than food’s reward value, which is larger when people look at food with a focus on taste. Moreover, four recent fMRI studies consistently found that, across BMI-groups, mindset affects not only selected portion size, but also the level of brain activity in response to high-caloric foods (i.e. prefrontal cortex in the health mindset, and orbitofrontal cortex in the hedonic mindset) (Bhanji & Beer, 2012; Hare et al., 2011; Hege et al., 2018; Veit et al., 2020). Taken together, neuroimaging results demonstrate that, while viewing high-caloric foods, the level of brain activity in self-control and reward-related networks is influenced by the current mindset.

Finally, evidence also suggests that the *power* of mind(set) on food perception extends to hormonal and behavioral effects. For example, it was shown that the ghrelin level (hunger hormone) was responsive to whether an ‘indulgent’ vs. a ‘health’ labelled milkshake was expected and consumed, whereas, in fact, the exact same milkshake was consumed in both conditions (Crum et al., 2011). The so called ‘food-label effect’ on foods (or drinks) consumption was observed in several studies (Crum et al., 2016; Crum & Zuckerman, 2017; Girz et al., 2012; Gustafson & Zeballos, 2020; Lim et al., 2018; Sullivan et al., 2015; for reviews see: Cecchini & Warin, 2016; Nikolaou et al., 2015). Furthermore, food-label studies indicate that ‘calorie’ labels on food promote dietary self-control, encouraging people to make healthier food choices (e.g., Hall et al., 2014; McCrickerd et al., 2016). Thus, by inducing a specific mindset, researchers minimize the risk of *mind-wandering* by participants. That is, the risk that, in absence of instructions (e.g., passive viewing) and/or a proper mindset manipulation, participants’ thoughts would frequently and repeatedly switch between hedonic and health while looking at high-caloric palatable foods. This scenario is undesirable as it complicates not only the interpretation of results, but also negatively affects internal validity and reproducibility of results.

Taken together, the effect of mindset, might help to explain the inconsistencies in the literature on AB for food (Alblas et al., 2020; Field et al., 2016a; Hagan et al., 2020; Anne Roefs et al., 2015; Werthmann et al., 2015). Therefore, the aim of the present study is to investigate the effect of the current mindset and BMI on AB for food and food intake. It is predicted that the power of food distractors to capture attention and eye-movements will be enhanced in the hedonic vs. the health mindset, mostly in people with obesity. Furthermore, previous findings show that the role of goal-driven and stimulus-driven attention, that is voluntary or involuntary control, depends on the moment in time in which selection takes place. That is, slow onset eye-movements (i.e., late first saccade latency) are more strongly driven by top-down strategy compared to fast onset eye-movements (i.e., early first saccade latency) which are automatically driven by physical characteristics of the stimuli (van Zoest et al., 2004, 2010). Using *time-course analysis (or bin analysis)*, we investigate whether the effects of mindset take time to establish, therefore being visible only at a later stage of oculomotor selection, on trials with a relatively late first saccade latency (Heimler et al., 2015; Hickey et al., 2010; van Zoest et al., 2010; Van Zoest et al., 2012; Van Zoest & Donk, 2008). Finally, for the bogus taste test, we expect that more snack food will be consumed after the hedonic mindset manipulation as compared to the health mindset manipulation, and that this effect will be more pronounced for people with obesity.

## Method

### Experimental Design

The results of this study are analysed in a 2 (Mindset: health vs hedonic) × 2 (BMI group: obese vs healthy-weight-category BMI) × 2 (distractor type: food vs neutral) mixed ANOVA.

### Participants

Potential participants were screened on dietary restraint (Restraint Scale; Herman & Polivy, 1980) and self-reported body mass index (BMI; kg/m2) via an online questionnaire sent via email at least one week before participation. Participants were recruited via paper flyers, online advertisements, local newspaper posts, and participant recruitment service (Link2Trials). Exclusion criteria were checked in a phone interview and included: pregnancy, psychiatric disorders, history of neurological and/or gastric surgical interventions.

In total, 82 participants volunteered for the study: 42 with healthy-weight-category BMI (BMI range: 18.5 –24.9) and 40 with obesity (BMI range: ≥ 30). Of this group, seven participants did not show up for the second session or did not make it within approximately one month from the first session (*n* = 3 with healthy-weight-category BMI, *n* = 4 with obesity). Two participants (both with obesity) were excluded because they did not eat chocolate during the bogus taste test, and one participant was excluded because the eye-tracking system crashed unexpectedly, and one participant (healthy-weight-category BMI) was excluded due to system calibration failure. Finally, five participants were excluded due to an insufficient number of valid trials, (see the exact criteria in the *eye-movement data* section below). The final sample consisted of 66 female participants: 35 with a healthy-weight-category BMI and 31 with obesity (see ***Table 1***). This sample size is in line with previous studies using the ASP in a similar design (e.g., Adam & Serences, 2021; Awh et al., 2012; Munneke et al., 2015; Theeuwes et al., 1998; Theeuwes & Belopolsky, 2012; Theeuwes & Godijn, 2002).

**Table 1:** Participant characteristics.


VARIABLE	HW (*n* = 35)			OB (*n* = 31)			*t*(64)	*p*
	
	M	SD	RANGE	M	SD	RANGE		

Age	43.3	9.0	27–54	43.3	9.3	28–55	0.34	.97

BMI	22.0	1.8	20.3–24.2	37.0	5.2	30.5–44.8	16.05	< .000

RS	10.4	5.0	2–23	17.5	4.5	10–26	6.17	< .001


*Note*: BMI = Body Mass Index, RS = Restraint Scale (Herman & Polivy, 1980). **Abbreviations:** HW = healthy-weight-category BMI; OB = obese BMI.

Notably, all participants reported normal (*n* = 64) or corrected-to-normal (*n* = 2) vision. Finally, the allocation of participants to the group with healthy-weight-category BMI or the group with obesity was based on the BMI score (kg/m^2^), measured in the laboratory at the end of the second session. Prior to the start of testing participants, the present study was pre-registered on ‘AsPredicted’ (*https://aspredicted.org/blind.php?x=6bb5fc*) and approved by the faculty’s ethical review board (ERCPN code: ECP- 159_15_12_2015 S8).

### Procedure

One week before the day of testing, the participant completed the online screening questionnaire. No participant was excluded based on the online questionnaire. On the day of testing, the participant was asked to refrain from eating and drinking two hours’ prior the start of the session (except water), and to eat something small (e.g., a sandwich and a small snack or piece of fruit) exactly two hours before the start of the session. In the laboratory, the participant read and signed the informed consent form, and completed the hunger assessment questionnaire. Right after, the participant received instructions about the visual search task, the eye-tracking equipment was calibrated and validated, and one block of practice trials (30 trials) began. Next, just before the start of the first block of the visual search task, the video-clip (mindset induction) was played, immediately followed by the mindset manipulation check. Next, the eye-tracking equipment was quickly calibrated and validated (same procedure, much quicker after the first time), and the participant completed the first block of the visual search task (*n* = 156 trials). After a brief break, the mindset video-clip and manipulation check were repeated, the eye-tracking system was recalibrated, and the second block of trials (*n* = 156) began. Subsequently, the participant was accompanied to a different room, where the bogus taste test took place. This concluded the first session, and the second session was scheduled.

To avoid interference between the two mindset manipulations, the two sessions were scheduled approximately 5 weeks apart. The order of the two mindset manipulations was counterbalanced across participants. At the end of the second session, the RS questionnaire was administered again, and the participant was asked to write down what they thought was the goal of the study. After reviewing all the given answers, nobody guessed the exact hypothesis of the present study. In particular, nobody pointed at the expected effect of the mindset manipulation on both the visual search task and food intake. Finally, height and weight were measured to compute BMI, and the participant received a €25 voucher.

### Questionnaires

#### Dietary Restraint

The dietary Restraint Scale (RS) (Herman & Polivy, 1980) is an 11-item questionnaire that assess weight concerns, weight fluctuations, and dieting attempts, and was administered to characterize our sample. This was administered twice: approximately one week before the first session (online; Qualtrics), and at the end of the second session (on paper). Approximately five weeks elapsed between the two measurements, and the average score is reported (***Table 1***). Cronbach’s alpha was high for both measurements (α = .81, and α = .83). As expected, people with obesity scored higher on dietary restraint than did people with a healthy weight-category BMI (see ***Table 1***).

#### Hunger Assessment

To control hunger level, participants were asked to eat something small (e.g., a sandwich accompanied by a small snack or piece of fruit) exactly two hours before the start of the study, and to refrain from eating until the start of the session (they could drink water only). At the beginning of each session, we provided participants with a three-item questionnaire: the first item assessed the time that elapsed since the last eating moment (in minutes), the second item asked participant to describe what they ate last, and the third item assessed participants’ (self-reported) hunger on a 100-mm VAS ranging from 0 (not hungry at all) to 100 (very hungry).

### Mindset Manipulation

Two brief video-clips (approximately 1-minute long) were created in Adobe Premiere Pro, based on audio-visual material from the Internet. In each session, either the hedonic or the health video-clip was presented, prior to the start of each of the two blocks of the Additional Singleton paradigm. Participants were asked to wear a pair of headphones (Jabra Move) for better immersion into the video-clip. The content of the two video-clips was adjusted to the age group of the participants. That is, the health mindset video-clip included sports activities (Nordic walking, yoga, aqua-gym), mixed with scenes depicting the preparation of healthy food recipes (for instance, grilled salmon with vegetables, salads). The hedonic mindset showed families and small groups of friends dining on a boat in Venice (Italy), while a chef is illustrating the menu and serving the food. Mindset-fitting background music was added to each of the video-clips. The two video-clips can be found at the following links: hedonic mindset video-clip, *https://www.youtube.com/watch?v=QND7B9oe5K8&t=9s*; health mindset video-clip, *https://www.youtube.com/watch?v=kEx1fcUmlJw&t=2s*.

**Figure 1:**
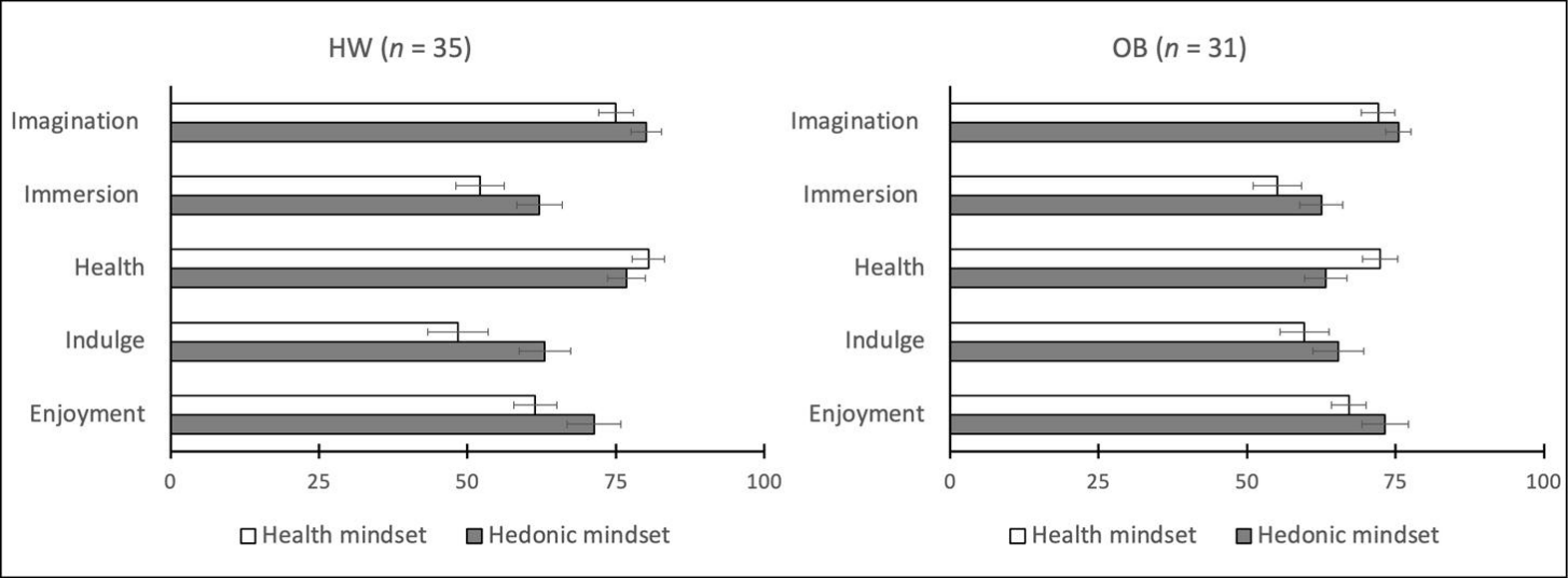
Mindset manipulation check results displayed per item and BMI-group (left graph: participants with healthy-weight-category BMI, right graph: participants with obesity). Error bars reflect 1 standard error of the mean in each direction. **Abbreviations:** HW = healthy-weight-category BMI; OB = obese BMI.

Video-clips were rated on 100-mm visual analogue scales (VAS) containing five items presented in a pseudo-random order^1^. The items asked: (a) To what extent could you imagine yourself in the movie? (b) To what extent did you feel immersed in the movie? (c) How inclined are you to choose healthy food at this moment? (d) How likely is it that you would indulge in tasty food at this moment? (e) How important is enjoying food to you at this moment? The VAS ranged from 0 (not at all) to 100 (very much). To assess the effect of the mindset manipulation (see ***Table 2***), five separate ANOVAs were conducted, one per item, with mindset (within subject) and BMI-group (between subject) as factors. Each item’s score is the average of the first and the second rating of the video-clip within the same session. No significant mindset × BMI effect was observed (all *F*s < 1.13, all *p*s > .20). The effect of mindset was significant in 4 of 5 ANOVAs, as described in ***Table 2*** and ***Figure 1***. As expected, participants scored higher on *Indulgence* and *Enjoyment* after the hedonic as compared to the health mindset induction. Similarly, participants scored higher on *Health* importance after the health mindset induction than after the hedonic mindset induction. Moreover, *Immersion* was higher and *Imagination* slightly higher for the hedonic than for the health mindset induction.

**Table 2:** Main effect of mindset per item of the mindset manipulation check. Reported Cohen’s *d* reflect the mindset manipulation’s effect size across BMI-groups.


ITEMS	*F* (1,64)	*p*	*d*

Imagination (a)	3.91	.052	.28

Immersion (b)	10.50	.002	.39

Health (c)	7.74	.007	.33

Indulge (d)	9.30	.003	.39

Enjoyment (e)	10.02	.002	.38


### Experimental Paradigm

**Figure 2:**
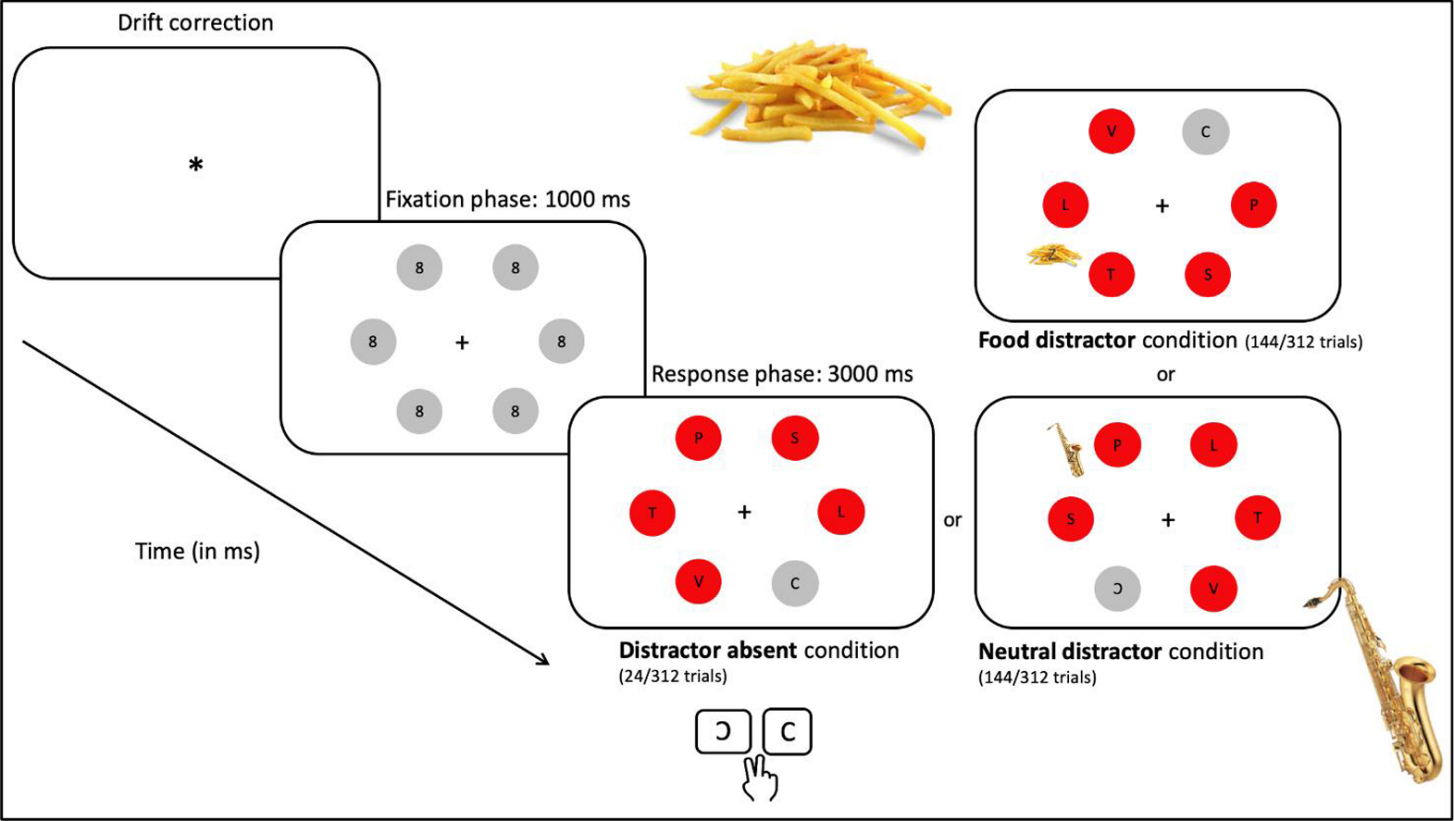
Graphical representation of the modified Additional Singleton paradigm (ASP). Distractors were presented at either 150° (top-right search-display) or 90° (bottom-right search-display) separation from the target.

A modified version of the *Additional Singleton Paradigm* (Theeuwes, 1991, 1992; Theeuwes et al., 1998) was used. At the start of each trial, a display of six grey circles (4° in diameter) was presented. Each circle contained a small figure-eight pre-mask, and circles were equally spaced on an imaginary circle (with a radius of 12.6°). A black fixation cross (0.4° of visual angle) was presented in the middle of the imaginary circle (center of the screen). The participant was instructed that eye-movements were necessary to correctly identify the letter inside the circles. Each item in the display was presented at 5° from the neighboring one. After 1s, all circles except one changed color to red, and the pre-masks inside the circles changed to letters. The target item (the grey circle) contained either a ‘C’ or a ‘reversed C’, whereas inside the non-target items (the red circles) the following letters were displayed: S, E, H, P, F, U (just like Theeuwes et al., 1998). On 90% of the trials, an additional item (musical instrument or food picture) appeared in the display, as a distractor, and was either separated by 90° or 150° from the target. As soon as the search display changed colors, the participant was instructed to make a quick and accurate eye-movement to the only grey circle, and to indicate if the circle contained a ‘C’ or a ‘reversed C’ by a button press on a button box. The search display was presented on screen for 3 s (maximal response time). Between trials, a blank screen was displayed for 500 ms (the inter-trial interval). Manual response times, response accuracy, and eye-movements were recorded. See ***Figure 2***, below, for a graphical representation of the experimental paradigm.

### Stimuli

In total, 70 picture stimuli were used: 35 pictures of musical instruments (neutral stimuli), and 35 pictures of high-caloric foods (size: 3.7° in diameter, resolution: 96 pixels/inch). Importantly, pictures of (high-caloric) foods and musical instruments were visually matched in color and size. All stimuli were retrieved from: Internet (iStockphoto), a database in our laboratory, and from an open source database of the Eating Behavior Laboratory, Salzburg University (Blechert et al., 2014, 2019).

### Trial Type

In total, each session consisted of 312 trials, preceded by 30 practice trials. In the practice phase, instead of food or a musical instrument, an additional red dot appeared sometimes on screen, with an abrupt onset. During the actual experiment, on 90% of the trials (288 trials), concurrent with the color change, a distractor appeared at either 150° or 90° of separation from the target. This picture was task-irrelevant and was either a high-caloric food (food distractor: 144 trials) or a musical instrument (neutral distractor: 144 trials). On the remaining 10% of the trials, no additional picture was presented (distractor absent: 24 trials). The 312 trials were divided in two equal blocks of 156 trials each. The computer task took approximately 30 minutes to complete.

### Eye-movements Measurement

Testing took place in a sound-attenuated, dimly lit room. Throughout the visual search task, the participant was seated on a chair next to a tower-mount frame and could rest the head on a chinrest. The monitor was placed exactly at 57 cm distance from the participant’s eyes. A 32-inch Philips monitor with a resolution of 1920 × 1080 pixels and a 100-Hz refresh rate was used (with these settings, 1° of visual angle equals to 1 cm). Eye-movements were registered with the Eyelink 1000 tower-mount system (SR Research Ltd., Canada), with 1.000-Hz temporal resolution, 0.01° gaze resolution, and a gaze position accuracy of 0.5°.

As for the calibration and validation procedure, the participant had to fixate nine calibration targets (black dots). The nine dots appeared on screen one at a time, in a 3 × 3 grid. The same procedure was repeated twice, one time for the calibration phase, a second time for the validation phase. As soon as one dot was fixated stably by the participant, the following was displayed in a different location.

### Bogus Taste Test

Seated at a table, the participant was presented with four glass bowls of the same size (13.5 cm in diameter), each filled with a different high-caloric food (on average 5.25 kcal/gr). Two bowls contained crisps (salted and paprika, on average 557.50 gr per bowl), whereas the other two contained chocolate (M&Ms, on average 854.16 gr per bowl; Maltesers, on average 603.67 gr per bowl). The participant was instructed to focus on the foods’ taste and to rate the foods. The participant was informed that they could taste as much as they would like. Four questionnaires (one per type of food) were positioned right next to the respective bowl. These questionnaires consisted of six items, for example: How appealing do you think the crisps/chocolates look?; How tasty do you find the crisps/chocolates? The items were answered on 100-mm VAS ranging from 0 (not at all) to 100 (very much). If the participant was done before the researcher returned to the room, the participant was instructed to taste some more food, without changing any answers. Each participant was given exactly 10 minutes to perform the bogus taste test. Importantly, before and after the bogus taste test, the food was weighed using a precision balance (PB3002 Mettler Toledo) to determine food intake (in grams) per food type. Finally, grams were converted to kilocalories, and the total number of kilocalories consumed was computed for each session and for each participant.

### Data Analysis

Data were analyzed in 3-way mixed ANOVAs, including one between-subjects factor (BMI: healthy-weight-category vs. obese) and two within subject factors (mindset: hedonic vs. health, distractor type: neutral vs. food).

*Manual response latencies*: In total, across both sessions, 3.55% of the trials were discarded from manual response latency analyses, either because the participant did not press a response key at all (0.03%) or pressed the wrong response key (1.42%). Moreover, manual response latencies were considered outliers and excluded from analyses if: (a) they were faster than 100 ms (0%), (b) they were slower than 2000 ms (0.50%) (Theeuwes et al., 2003), and next if (c) they deviated more than 3 SD in either direction from each participant’s mean (1.60%) (Castellanos et al., 2009; Mogg et al., 1998; Werthmann et al., 2011).

*Eye-movement data: Invalid trails were excluded from the analysis if: (a) the f*irst saccade latency was quicker than 80 ms (anticipatory saccades, 0.67%) or longer than 600 ms (2.23%) (van Zoest & Donk, 2005, 2008; van Zoest et al., 2004; Theeuwes et al., 2003); (b) eye-movements starting outside the interest area set around the fixation cross (2.5° of visual angle degree, the radius) (3.88%). In total, this led to the exclusion of 6.78% of the total trials. Based on the above-mentioned exclusion criteria, participants not reaching at least 2/3 of valid trials (208 trials of 312, excluding practice phase) were excluded from the analyses (both eye-movements and RTs). This led to the exclusion of five participants (3 with healthy-weight-category BMI, 2 with obesity). Finally, an interest area of 6° visual angle (the radius) was set around the target and the distractor and based on this area the following dependent variables were extracted, per trial type: (a) Percentage of trials with a fixation on the distractor (i.e., the number of trials on which the distractor was fixated, divided by the number of trials on which the distractor was present). To define fixations on the distractor, a circumference of radius 6 visual angle degrees was set around the item (about 6 cm in our setting). (b) Duration of the first fixation on the distractor, (c) total dwell-time on the distractor, and (d) first saccade latency (that is, the elapsed time between the onset of the stimulus and the start of the eye-movement).

### Additional analyses

#### Time-course analysis

For each participant, a distribution of the first saccade latencies (i.e., time that elapses before the eye-movement is initiated) was extracted. Each participant’s distribution was divided into three tertiles (called *bins*) based on the latency of the first saccade: quick, medium, and slow. For two dependent variables (manual reaction times, and percentage of trials with a fixation on distractor) the averages were computed per bin as input for the time-course analyses. Total dwell time and duration of the first fixation (on the distractor) could not be analyzed, per bin, due to insufficient amount of data in each bin.

#### Correlations Analyses

In addition, several correlations were computed. It was tested if measures of AB for food correlated with food intake and with dietary restraint. We also tested if mindset manipulation effectiveness correlated with food intake during the bogus taste test. Notably, each correlation was computed per mindset (for the health and hedonic mindset separately) and across mindsets (averaged across health and hedonic mindset). For each AB variable, the *bias score* was computed, per and across mindsets, by subtracting the mean response of trials with a neutral distractor from the mean response of trials with a food distractor. A positive bias score indicates an attention bias towards food, and a negative bias score indicates an attention bias away from food.

## Results

### Hunger level assessment

Overall, across BMI-groups and sessions, participants reported relatively low hunger level right before the start of each session (*M* = 22.29, *SD* = 13.33). Similarly, across BMI-group and sessions, all participants complied with the eating instruction, as evidenced by the elapsed time between the last meal and the start of the session (M = 133.44, SD = 16.60). Hunger levels did not differ significantly between BMI-groups or mindsets, all *F*s < 1.34, all *p*s > .25 (see ***Table 3*** for mean and SD).

**Table 3:** Mean and SD of the hunger check variables per BMI-group and per mindset.


HUNGER CHECK VARIABLES	HW (*n* = 35)		OB (*n* = 31)	
	
	HEALTH MINDSET	HEDONIC MINDSET	HEALTH MINDSET	HEDONIC MINDSET
	
	M (SD)	M (SD)	M (SD)	M (SD)

Hunger level (VAS)	21.25 (14.91)	20.20 (14.56)	23.90 (12.24)	24.80 (13.07)

Last eating moment (min)	134.14 (22.63)	134.28 (18.67)	130.64 (16.52)	137.09 (21.32)


**Abbreviations:** HW = healthy-weight-category BMI; OB = obese BMI.

### Behavioral results

Response accuracy (%) and manual response latencies were analyzed in 2 (mindset: health vs. hedonic) × 2 (BMI group: obese vs. healthy-weight-category BMI) × 2 (distractor type: food vs. neutral) mixed ANOVAs.

*Response accuracy*. No significant effects were observed, all *F*s (1,64) < 3.45, all *ps* > .067 (see ***Table 4*** for means and SDs).

**Table 4:** Response accuracy per condition of the design.


	HW (*n* = 35)				OB (*n*= 31)			
	
	HEALTH M		HEDONIC M		HEALTH M		HEDONIC M	
			
	NEUTRAL D	FOOD D	NEUTRAL D	FOOD D	NEUTRAL D	FOOD D	NEUTRAL D	FOOD D
			
	M (SD)	M (SD)	M (SD)	M (SD)	M (SD)	M (SD)	M (SD)	M (SD)

Accuracy (%)	98.83 (1.40)	98.73 (1.19)	98.79 (1.01)	99.08 (0.88)	98.54 (1.55)	98.43 (1.30)	98.01 (2.43)	98.33 (1.48)


**Abbreviations:** HW = healthy-weight-category BMI; OB = obese BMI; *D* = distractor type; *M* = mindset; BMI = Body Mass Index.

**Figure 3:**
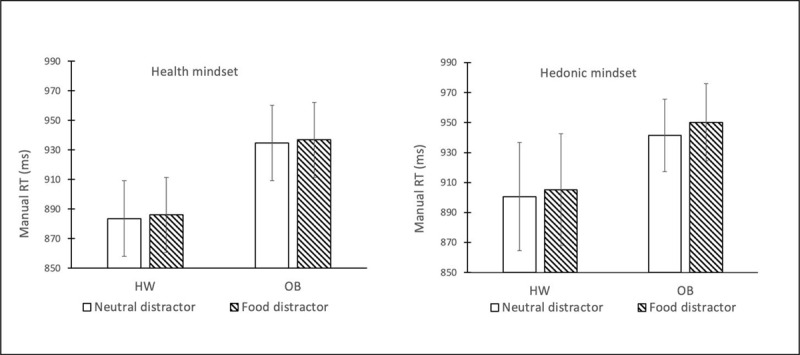
Average manual response latency per condition of the design. Error bars reflect 1 standard error of the mean in each direction. **Abbreviations:** HW = healthy-weight-category BMI; OB = obese BMI.

*Response latencies*. Overall, response latencies were faster in the distractor absent condition (*M* = 893.34, *SD* = 142.94) than in the distractor present conditions (*M* = 907.31, *SD* = 150.49), *t*(65) = 3.61, *p* = .001, *d* = .09. Notably, average response latency was slower with a food distractor than with a neutral distractor, as evidenced by a significant main effect of distractor type, *F*(1,64) = 5.58, *p* = .02, *η_p_*^2^ = .08. The observed main effect was not significantly qualified by either mindset, *F*(1,64) = 0.66, *p* = .42, *η_p_*^2^ = .01 or BMI, *F*(1,64) = 1.50, *p* = .22, *η_p_*^2^ = .02, nor was the distractor type × mindset × BMI interaction significant, *F*(1,64) = 0.39, *p* = .53, *η_p_*^2^ = .01. See ***Figure 3*** for relevant means and SDs.

### Eye-movement results

Eye-movements were analyzed in 2 (mindset: health vs. hedonic) × 2 (BMI group: with obesity vs. with healthy-weight-category BMI) × 2 (distractor type: food vs. neutral) mixed ANOVAs for each of the three dependent variables. All relevant means and SDs per condition of the design can be found in ***Table 5***. Unexpectedly, no significant main effects or interaction effect were observed in any of the ANOVAs, all F < 2.61, all p > .11 (see ***Table 5*** for all statistics relevant to the hypotheses).

**Table 5:** Eye-movements’ variables.


VARIABLES (*n* = 3)	HW (*n* = 35)				OB (*n* = 31)				D		D*M		D*BMI		D*M*BMI	
	
	HEALTH M		HEDONIC M		HEALTH M		HEDONIC M									
			
	NEUTRAL D	FOOD D	NEUTRAL D	FOOD D	NEUTRAL D	FOOD D	NEUTRAL D	FOOD D								
							
	M (SD)	M (SD)	M (SD)	M (SD)	M (SD)	M (SD)	M (SD)	M (SD)	*F*(1,64)	*p*	*F*(1,64)	*p*	*F*(1,64)	*p*	*F*(1,64)	*p*

Percentage of trials with a fixation on the distractor	9.54 (14.16)	10.03 (17.34)	9.40 (15.30)	9.09 (17.34)	10.83 (11.85)	11.12 (12.09)	14.15 (18.40)	13.85 (18.05)	0.09	0.92	1.47	0.23	0.01	0.91	0.03	0.86

Duration (in ms) of the 1^st^ fixation on the distractor (a)	97.70 (35.36)	86.96 (37.18)	99.27 (33.34)	97.09 (35.02)	108.63 (35.40)	104.72 (32.66)	105.64 (35.64)	107.01 (41.01)	2.61	0.11	1.22	0.27	1.18	0.28	0.07	0.79

Total dwell time (in ms) on the distractor (b)	113.55 (57.22)	102.30 (60.86)	113.00 (65.52)	118.42 (86.00)	134.19 (74.22)	128.98 (57.74)	131.92 (79.94)	127.23 (65.24)	0.59	0.45	1.25	0.27	0.04	0.84	1.10	0.30


*Note*: (a) and (b): reduced sample due to missing data, because some participants did not fixate the distractor. In total *n* = 54 participants out of *n* = 66, of which *n* = 27 with healthy-weight-category BMI and *n* = 27 with obesity. **Abbreviations:** HW = healthy-weight-category BMI; OB = obese BMI; *D* = distractor type; *M* = mindset; BMI = Body Mass Index.

### Bogus taste test results

**Figure 4:**
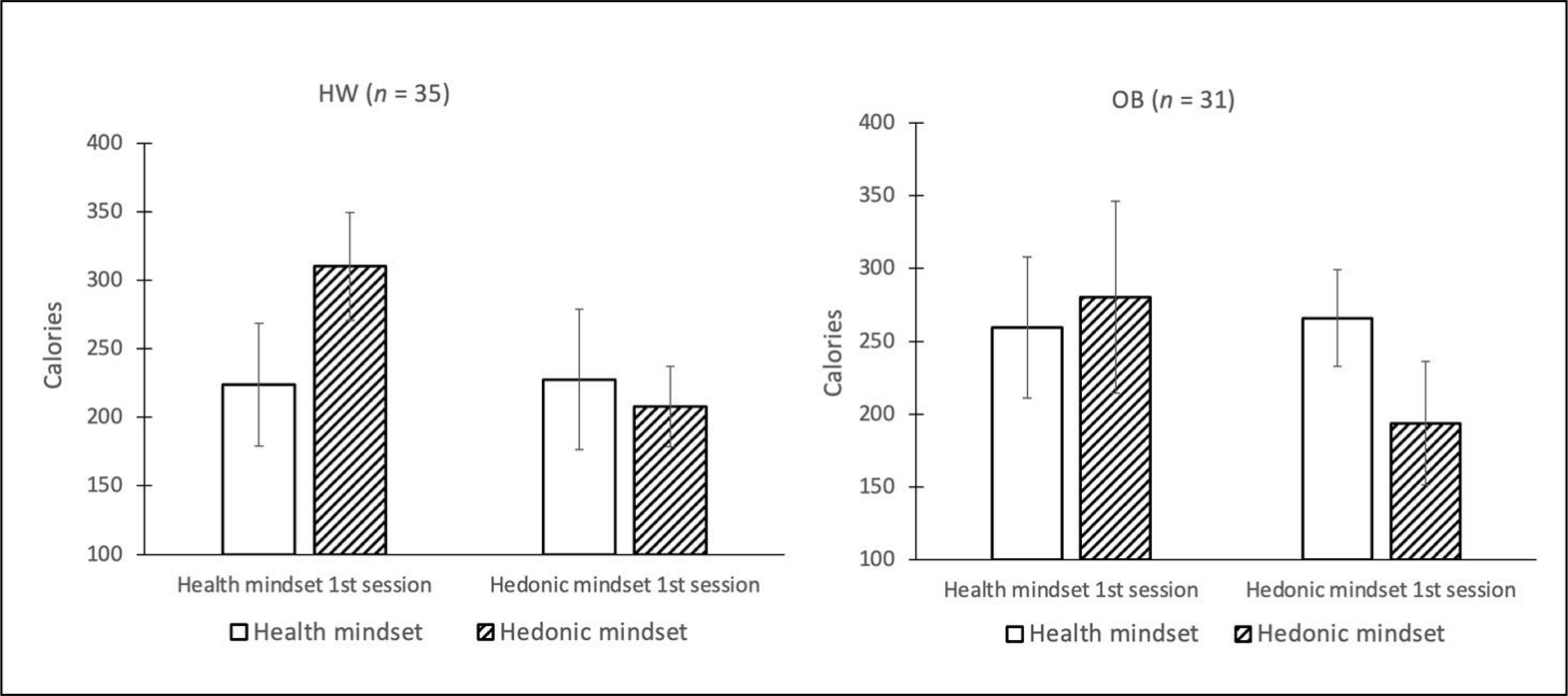
Food intake during bogus taste test per condition. Error bars reflect 1 standard error of the mean in each direction. **Abbreviations:** HW = healthy-weight-category BMI; OB = obese BMI.

Food intake (kcal) was analyzed in a 2 (mindset: health vs. hedonic) × 2 (BMI group: obesity vs. healthy-weight-category BMI) × 2 (session order: hedonic mindset first versus health mindset first) mixed ANOVA. *Session order* was included as a factor in the analysis to control for the so called *neophobia effect*. That is, participants need some time to get used to eating in the laboratory and therefore often eat less food in the first session than in later sessions (Guerrieri et al., 2007; Overduin & Jansen, 1997; Roefs & Jansen, 2004). The effect of mindset on food intake depended on session order, as evidenced by a session order × mindset interaction, *F* (1,64) = 9.51, *p* = .003, *ηp*^2^ = .13. Follow-up *t*-tests showed that participants consumed more food in the hedonic mindset compared to the health mindset when the health mindset was induced first, *t*(32) = 2.35, *p* = .025, *d* = .26. The effect was reversed, that is, participants consumed more food in the health mindset than in the hedonic mindset, when the hedonic mindset was induced first, *t*(32) = 2.06, *p* = .047, *d* = .28. In addition, for the health mindset, the difference between the two orders was not significant, *t*(32) = 0.12, *p* = .93, *d* = .03, whereas it was trend-significantly different for the hedonic mindset, *t*(32) = 1.99, *p* < .055, *d* = .49. Finally, no significant main effects of BMI: *F*(1,64) = 0.03, *p* = .86, *η_p_*^2^ = .004, mindset: *F*(1,64) = 0.05, *p* = .81, *η_p_*^2^ = .001 or mindset × BMI: F(1,64) = 3.34, p = .07, *η_p_*^2^ = .05 were observed (see ***Figure 4*** for M and SD).

### Additional analyses results

#### Time-course analysis results

To test whether an effect of mindset was present on trials with a relatively slow first saccade latency, a time-course (or bin) analysis was conducted. Trials were divided into fast, medium, and slow bins based on the first saccade latency. This type of analysis was performed on two dependent variables: manual response latency, and percentage of trials with a fixation on distractor. Data were analyzed in 2 (mindset: health vs. hedonic) × 2 (BMI group: obesity vs. healthy-weight-category BMI) × 2 (distractor type: food vs. neutral) × 3 (bin: fast, medium, slow) mixed ANOVAs.

**Figure 5:**
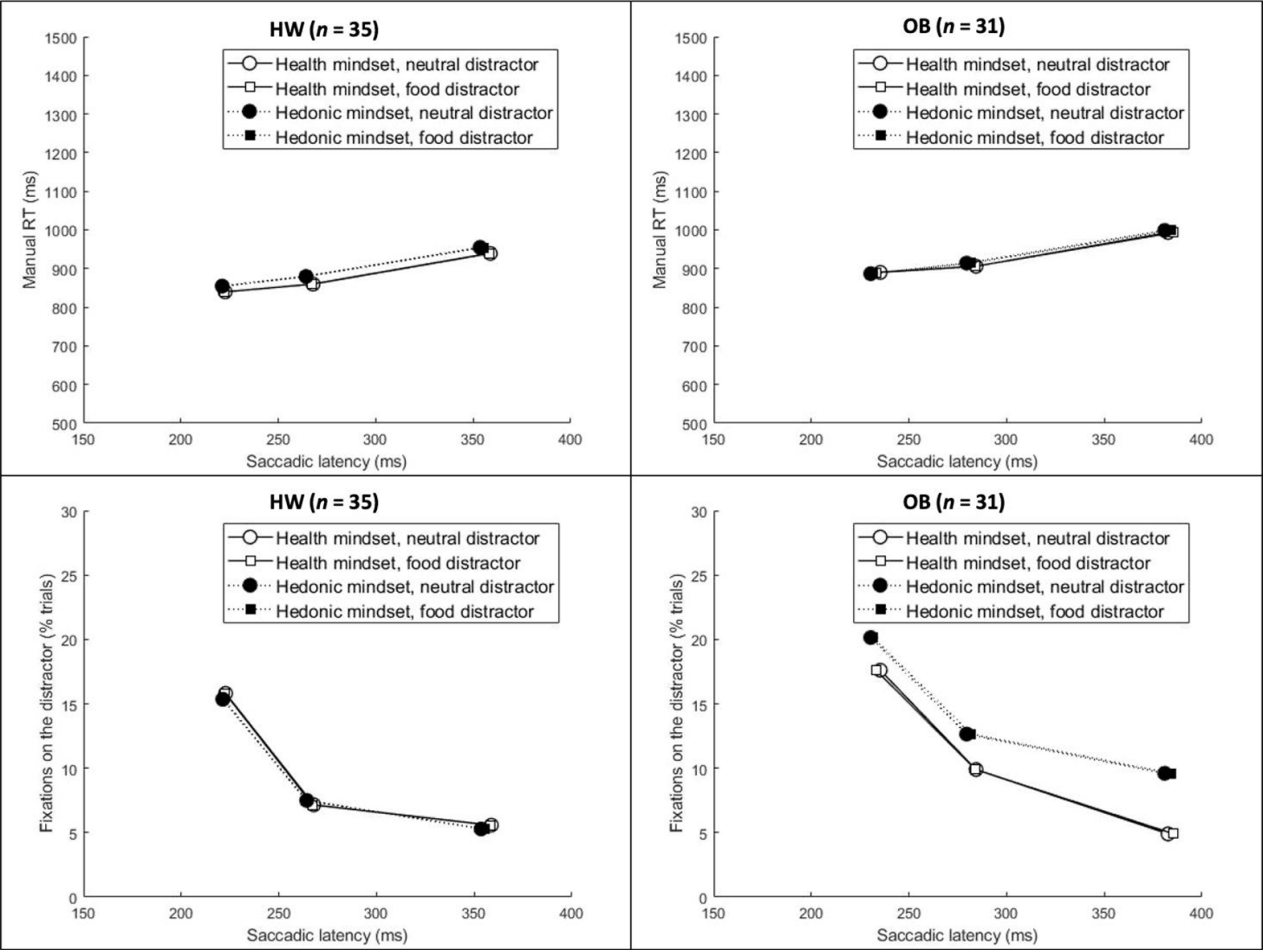
**Top panels:** Average manual RT as a function of first saccade latency, mindset, and distractor type. **Bottom panels:** The average proportion of trials with a fixation on the distractor as a function of first saccade latency, mindset, and distractor type. **Abbreviations:** HW = healthy-weight-category BMI; OB = obese BMI.

*Manual response latency*. In line with the first ANOVA on response latencies, a significant main effect of distractor type was observed, *F*(1,64) = 7.17, *p* = .009, *η_p_*^2^ = .10. Furthermore, response latencies were longer for slower bins, as evidenced by a main effect of bin, *F*(1,64) = 244.35, *p* < .001, *η_p_*^2^ = .79. None of the other effects reached significance, all *F*s (1,64) < 1.70, all *ps* > .19 (see ***Figure 5*** for relevant means and SDs).

*Percentage of trials with a fixation on distractor*. The percentage of trials on which the distractor was fixated was higher for the faster trials, as evidenced by a main effect of bin, *F*(1,64) = 49.50, *p* < .001, *η_p_*^2^ = .44. In addition, the percentage of trials with a fixation on the distractor was higher for the hedonic mindset than for the health mindset for participants with obesity only, specifically in the slow bin, as evidenced by a mindset × BMI group × bin interaction, *F*(1,64) = 3.11, *p* = .048, *η_p_*^2^ = .04. The hedonic mindset increased oculomotor capture of irrelevant distractors for participants with obesity, but not for participants with a healthy-weight-category BMI. As follow-up analyses, we computed the average for the two distractor types per bin and per mindset for each participant. For the participants with obesity, we compared the hedonic vs. health mindset per bin. None of these three *t*-tests were significant, fast: *t*(30) = 0.88, *p* = .38, *d* = .12; medium: t(30) = 1.11, *p* = .27, *d* = .17; slow: *t*(30) = 1.73, *p* = .093, *d* = .33. See ***Figure 5*** for relevant means and SDs.

#### Correlations between AB variables and food intake

In the analyses per mindsets, a trend-significant correlation between manual RTs (bias score) and food intake, in the health mindset only, was observed, *r*(66) = .23, *p* = .06. When computed across mindsets, one more trend-significant correlation was found between food intake and the percentage of trials on which the food distractor was fixated, *r*(66) = .24, *p* = .056. None of the other correlations, per mindset, all *rs*(66) < .20, all *p*s > .11, and across mindsets, and all *rs*(66) < .08, all *p*s > .84, reached (trend) significance.

#### Correlations between AB variables and dietary RS level

No correlations between AB variables (bias score) and dietary RS, computed per mindset, all *r*s < 0.17, all *p*s > .19, and across mindsets, all *r*s < 0.87, all *p*s > .48, were significant.

#### Correlations between mindset manipulation check variables and food intake

Finally, we tested whether the scores of the mindset manipulation check correlated with food intake. When computed per mindset, we observed four significant correlations with food intake. In the health mindset, food intake correlated negatively with the score on the *Health* item, *r*(66) = – .27 *p* = .03. In the hedonic mindset, food intake correlated positively with the *Indulge* item *r*(66) = .40, *p* < 0.001, the *Imagination* item, *r*(66) = .34, *p* = 0.005, and the *Immersion* item, *r*(66) = .29, *p* = .02. None of the other correlations computed per mindset reached significance, all *rs*(66) < .18, all *p*s > .17. When computing the correlations across mindsets, two correlations reached significance: a negative trend-significant correlation was found between food intake and the *Health* item, *r*(66) = – .24, *p* = .051. A positive correlation was found between food intake and the *Indulge* item, *r*(66) = .29, *p* = .016.

## Discussion

The aim of the present study was to unravel whether the current mindset (hedonic vs. health) in interaction with BMI (healthy-weight-category vs. obese) influences AB for high-caloric foods and food intake. Contrary to our hypothesis, neither mindset nor BMI affected AB for high-caloric foods. Only a general AB for food as reflected in response latencies was observed. Analyses of eye-movements did not result in any significant findings. The time-course analysis revealed increased oculomotor capture by the distractors (both food and neutral) in the hedonic mindset on slow trials, specifically for participants with obesity. The effect of mindset on food intake was partly in line with the hypothesis as participants consumed more food in a hedonic than in a health mindset, but only when the hedonic mindset was induced in the second testing session. Only one measure of AB for food (fixations on the distractor, % trials) correlated positively with food intake. The manipulation of mindset was successful, as evidenced by the manipulation check, as well as by the meaningful correlations in the expected direction between the scores on the manipulation check and food intake during the bogus taste test.

The observed lack of significant moderation of AB for food by BMI contradicts theories on AB for food as a trigger of overeating and as a characteristic of people with obesity (Barry et al., 2009; Batterink et al., 2010; Nijs, Franken, et al., 2010; Stice et al., 2013). However, the lack of moderation by BMI for AB for food is not a novelty in the literature (Doolan et al., 2014; Kemps et al., 2016; Loeber et al., 2012; Nijs, Franken, et al., 2010; Stamataki et al., 2019), adding further doubts if AB for food can be considered a characteristic of people with obesity (Field et al., 2016a; Hagan et al., 2020; Roefs et al., 2015; Werthmann et al., 2015). Whereas people with overweight and/or obesity have a positive energy-balance (e.g., Hill, 2006; Roefs et al., 2015; 2018; Webber, 2003), it might not be the case that all people with overweight show an AB for food. It may also be possible that AB for food contributes to a positive energy balance only in a subgroup. The current study shows that, independent of BMI, people show an AB for food (reflected in response latencies only). This has been reported before, albeit in a different type of paradigm, mostly the dot-probe task (e.g., Freijy et al., 2014; Loeber et al., 2012).

Moreover, contrary to our hypothesis, the induced mindset (either hedonic or health) did not affect AB for food either. Whereas the mindset manipulation successfully impacted some behaviors, as evidenced by differences between mindsets on subjective measures, food intake, and meaningful correlations with food intake, mindset did not have a significant impact on AB for food. These findings are partially in disagreement with earlier research on this topic (Werthmann et al., 2016), in which it was found that AB for food in a dot-probe task was attenuated in a health mindset, specifically for high restrained eaters. One reason for this non-significant finding could be attributed to the small-to-medium effect size of the manipulation on the manipulation check (on average, *d* = .35). However, this effect size is in line with other studies in which similar experimental manipulations were used (e.g., Franssen et al., 2020a; Roefs et al., 2006; Werthmann et al., 2016). Another reason could be that the induced mindset manipulation was neither task-relevant, nor repeated every trial, which may be a necessary precondition to observe effects on AB for food. In line with this, seven recent studies (Bhanji & Beer, 2012; Franssen et al., 2020; Hare et al., 2011; Hege et al., 2018; Veit et al., 2020; Kochs et al., under review; Pimpini et al., submitted) found that changing the perspective (health vs. taste) with which participants *look* at foods modulates value and choice-related brain response. Importantly, all these studies adopted a task-relevant mindset manipulation, present on every trial.

Another challenging finding of the present study is that AB for food, observed in manual response latencies, did not transfer to oculomotor performance. The disconnect between oculomotor performance and RT-based attentional bias is not unprecedented, and the relationship between attention and eye-movement is overall complicated (e.g., Hunt & Kingstone, 2003; Hunt et al., 2009).

Moreover, the overall percentage of oculomotor capture in the present study was relatively low compared to previous similar studies looking at distractor effects by means of eye-movements. For example, in Theeuwes et al. (1998), about 35-40% of the initial eye-movements were affected by the abrupt presentation of a salient distractor (Theeuwes et al., 1998). In studies in which the distractor is not an abrupt onset, but a static singleton, capture can similarly occur in about half of the trials, especially when saccades are triggered quickly following display presentation (e.g., van Zoest, Donk & Theeuwes, 2004). In contrast, the percentage of eye-movements that was disrupted due to the irrelevant onset distractors was much lower in the present study (on average, 11%). Thus, one reason for the low capture rate observed in the present study might be that it was relatively *easy* for participants to ignore the abrupt onset of the distractor because of the high visual dissimilarity between the target (grey circle) and the task-irrelevant distractor (either food or musical instrument) (Poiese et al., 2008). In support for this explanation, it has been empirically shown that irrelevant abrupt onsets produce robust capture effects, only when the visual search task is difficult (Gaspelin et al., 2017).

In addition, strong top-down control strategies during visual search may have been further strengthened due to trial-type proportion. That is, the distractor (either food or musical instrument) was presented in 90% of the total trials, likely encouraging distractor suppression. In other experiments using the ASP, the distractor was presented in 50% or 75% of all trials (e.g., Adam & Serences, 2021; Kelley & Yantis, 2009).

Clearly, the above-mentioned characteristics of the ASP point at the *habituation effect* as the culprit for the low oculomotor capture rate by the distractor. Habituation effect is defined as a steady decrement of response following repeated irrelevant stimulation (Turatto & Pascucci, 2016). It is therefore plausible that participants quickly learned to ignore the abrupt onset of the distractor which eventually led to a low oculomotor capture rate. In the literature, this effect has been extensively investigated (Bonetti & Turatto, 2019; Failing et al., 2019; Kelley & Yantis, 2009; B. Wang et al., 2019; B. Wang & Theeuwes, 2018). This said, additional analysis conducted on only the first half of the first block, showed a similar capture rate by the distractor (14% the subset of trials vs. 11% the total number of trials).

In the time-course analysis, we observed an effect of mindset on oculomotor capture across distractor types. That is, we found that in the hedonic mindset participants with obesity fixated more often on the distractor (both food and neutral) on trials with a slower saccade onset. This suggests that, when in a hedonic mindset, people with obesity were more distracted by the onset of an irrelevant distractor on these slower trials. As this was the case independent of the type of distractor, it suggests that increased distractibility in the hedonic mindset (especially on the slower trials) may reflect an overall deteriorated top-down control. Though, this explanation remains speculative.

The results of the bogus taste test were partly in line with our hypothesis, as we found that participants ate more food in the hedonic mindset than in the health mindset, but only when this hedonic mindset was induced in the second session, and effects were not moderated by BMI. It seems that the first session was dominated by the food *neophobia effect*, that is, getting used to eating in a laboratory setting (Guerrieri et al., 2007; Overduin & Jansen, 1997; Roefs & Jansen, 2004), whereas in the second session the neophobia effect faded, but only if the hedonic mindset was induced. So, the hedonic mindset might have boosted food consumption when induced in the second session, contributing to the reduction of food neophobia. Alternatively, the health mindset might have inhibited food consumption when induced in the second session, counteracting the reduction of the neophobia effect. Note that the same pattern of results was found in a previous study (Roefs & Jansen, 2004) in which higher intake was reported for drinks with a low-fat label (as compared to a high-fat label), only when the drink with the low-fat label was presented in the second session.

Finally, the observed correlations between AB for food and food intake are in line with earlier research with adults and children with obesity (Fokvord et al., 2015; Hardman et al., 2021; Smith et al., 2020; Werthmann et al., 2011). That is, it is generally found that more AB for food is related to increased food consumption, which suggests that AB for food may be sensitive to fluctuations in the motivational value of food (Field et al., 2016; Hardman et al., 2021).

Taken together, food captures attention – but not the eyes, independent of BMI and mindset. As AB for food was not more pronounced in people with obesity, this study casts further doubt on the idea that AB for food is a specific characteristic of people with obesity. As previous studies did observe effects of mindset on AB for food (Field et al., 2016a; Anne Roefs et al., 2015, 2018; van der Laan et al., 2017; Werthmann et al., 2016) and as there is evidence that fluctuations in AB for food are increased with a higher BMI (Liu, Nederkoorn, et al., 2019; Liu, Roefs, et al., 2019), it would make sense for future studies to test effects of stronger, preferably task-based, manipulations of mindset on AB for food. Future studies would also benefit from adapting the Additional Singleton paradigm as to increase the capture rate by the distractor. For instance, using the same paradigm, a shape-singleton visual search could be implemented instead of a color-singleton search task. That is, while a color-singleton target implies quick and automatic parallel search (so-called pop-out effect), a shape-visual search would require longer and more cognitively demanding serial search, leaving some room for distraction.

### Future Research Directions

The findings of this study represent a solid starting point for future research on AB for food. To improve the paradigm, future studies could refine visual search (i.e., shape instead of colour-singleton search), and introduce a more equal trials’ proportion between trials with and without distractor. Moreover, future studies could tailor the food stimuli based on individual preferences and frequency of consumption. Future studies could also strengthen the mindset manipulation, by making it task-related and – if possible – repeated on every trial. Finally, the study of AB for food could be extended to male participants, and include different types of food restrictions (i.e., prevent weight gain, weight loss dieting, and weight suppression) and eating styles (Chen et al., 2021; Lowe & Levine, 2005).

## Data Accessibility Statement

Raw data and codes of this study can be viewed clicking on the following link: *https://osf.io/swhfv/?view_only=2bc84f66a8804b768425cfa481699fe4*.

## Footnotes

Items (a) and (b) were always presented first and second, whereas the order of the remaining three items was randomized per participants and per session (within session, between the first and second assessment, the order remained the same).
